# The Importance of SGLT-2 Inhibitors as Both the Prevention and the Treatment of Diabetic Cardiomyopathy

**DOI:** 10.3390/antiox11122500

**Published:** 2022-12-19

**Authors:** Klaudia Kowalska, Piotr Wilczopolski, Dominika Buławska, Ewelina Młynarska, Jacek Rysz, Beata Franczyk

**Affiliations:** 1 Department of Nephrocardiology, Medical University of Lodz, ul. Zeromskiego 113, 90-549 Lodz, Poland; 2 Department of Nephrology, Hypertension and Family Medicine, Medical University of Lodz, ul. Zeromskiego 113, 90-549 Lodz, Poland

**Keywords:** diabetes, insulin resistance, diabetic cardiomyopathy, SGLT2is, heart failure, oxidative stress, mitochondria, NADPH oxidase, AGEs, lipotoxicity

## Abstract

According to the 2021 report of the International Diabetes Federation (IDF), there have been approximately 573 million cases of type 2 diabetes mellitus (T2DM) among adults, which sets the disease as a major concern in healthcare worldwide. The development of T2DM is strongly promoted by unhealthy lifestyle factors associated with urbanization and western civilization. The disease is associated with a broad list of systemic complications that can result in premature death, disability and significantly reduced quality of life. The most dramatic in their consequences are cardiovascular complications of T2DM. Our work focuses on one such complication that is specific for diabetes, named diabetic cardiomyopathy (DC). In this condition cardiac dysfunction occurs despite the absence of underlying hypertension, coronary artery disease and valvular disease, which suggest a leading role for metabolic disturbances as a cause. We aimed to establish the role of relatively new hypoglycaemic drugs that have taken the medical world by storm with their broad pleiotropic effects—SGLT-2 inhibitors—in the prevention and treatment of DC at any stage.

## 1. Introduction

With the progress of civilization, there have been profound changes in the lifestyle and diet of citizens of western countries. These lifestyle and dietary patterns have been accompanied by the emergence of new, non-infectious, degenerative conditions characteristic for this part of the world, which are known as civilization diseases [1]. Diseases that have plagued the Western population include obesity, insulin-resistance leading to type 2 diabetes (T2DM), cardiovascular and autoimmune diseases, dyslipidaemia and cancer. As these conditions are interrelated and share common mechanisms and pathways, they often present in parallel such as in metabolic syndrome X [2,3,4]. What civilization diseases also have in common is the occurrence of systemic complications that can result in lifelong disability, significant reduction in quality of life as well as an increase in treatment expenses that have become a burden not only for the patients but also for the global economy [5,6]. A disease that consumes an enormous amount of funding is T2DM. According to a 2017 report by the Polish National Health Fund, the treatment of diabetes and its complications have consumed 6073 million Polish zlotys. The 2017 report indicates that the average cost per patient with diabetes is 82.5% higher than for a patient of the same sex and age but without type 2 diabetes, as diabetic patients are more prone to other conditions, and the course of their comorbidities tends to be severe [7].

Cardiac complications are the leading cause of death among diabetics. Insulin resistance and elevated glycaemic levels predispose diabetics to macro- and microvascular complications, which manifest mainly as coronary artery disease (CAD) that can lead to heart failure (HF). However, type II diabetes can cause heart failure regardless of the presence of hypertension and coronary or valvular defects [8]. This condition is termed diabetic cardiomyopathy (DC) and was identified in 1972 by Robert Shirley. DC features impair cardiomyocyte function due to impaired insulin myocardial signalling, mitochondrial dysfunction, defective sarcoplasmic reticular calcium transport, increased oxidative stress and abnormal microcoronary circulation, which may result in fibrosis, hypertrophy, impaired systolic function and heart failure [8,9,10,11]. The prevention of complications and slowing down the progression of the disease is the most cost-effective and beneficial therapeutic strategy for patients. Therefore, there is a need to optimise pharmacotherapy in the early stages of the disease using pleiotropic anti-diabetic agents such as sodium–glucose transporter-2 inhibitors (SGLT-2is), also known as flozins. Representatives of SGLT-2is, such as empagliflozin and dapagliflozin, have conquered the world of internal medicine research not only with their hypoglycaemic properties but also with their cardio and nephroprotective effects ever since the results of the EMPA-REG OUTCOME, EMPEROR-REDUCED, EMPEROR-PRESERVED, DAPA-HF, DELIEVER and DAPA-CKD trials have been published [12].

In this article, we aim to review the recent literature on diabetic cardiomyopathy, SGLT-2i and the role of flozins in the prevention and management of this disease.

## 2. Type 2 Diabetes Mellitus and Diabetic Cardiomyopathy

T2DM is one of the most common metabolic disorders worldwide and the most frequent of all types of this disease (more than 90% diabetes cases are T2DM). The International Diabetes Federation (IDF) reported that in 2019, 463 million adults aged between 20 and 79 years old suffer from diabetes, with most cases prevalent between 40 and 59 years old. According to the mentioned report, T2DM and its complications are a leading cause of death at 4.2 million people. Despite the common occurrence of the disease worldwide, an estimated 30% of individuals with T2DM are underdiagnosed and are therefore untreated [13,14,15,16].

Type 2 diabetes mellitus is a metabolic disorder which is caused by impaired insulin secretion, insulin resistance (IR) or a combination of both, which leads to dysregulation of carbohydrate, lipid and protein metabolism. In the early stage of the disease, insulin level can be normal or even increased, which changes in later stages, especially in poorly controlled cases, when insulin secretion becomes impaired. Insulin secretion reduction occurs due to both the loss of β-cells and dysfunctional molecular pathways. Factors that contribute to metabolic dysregulation in β-cells are: ageing, genetic abnormalities, lipotoxicity, glucotoxicity, reactive oxygen stress, incretin hormone (glucagon-like peptide 1 (GLP1) and gastric inhibitory polypeptide (GIP)) resistance and/or deficiency, IR leading to β-cells stress, hypersecretion of islet amyloid polypeptide (IAPP) and activation of inflammatory pathways. On the other hand, insulin resistance contributes to the decrease in glucose uptake in liver, muscles and adipose tissue, whereas it increases gluconeogenesis in liver and lipolysis in adipocytes [14,15]. Many of above-mentioned risk factors present in T2DM are known as cardiovascular risk factors. On this account, T2DM itself is considered a well-established risk factor for cardiovascular disease (CVD). Diabetic vascular disease is associated with a twofold higher incidence of CAD and stroke as well as with an increase in the risk of developing heart failure by two to eight times [17]. CV complications are responsible for approximately 50% deaths among patients with T2DM. On the other hand, diabetes mellitus was associated with an 18% increase in cardiovascular mortality [18,19]. Men with diabetes are twice as likely to develop heart failure than non-diabetic men. Diabetic females are even more prone to serious cardiovascular complications, as they are five times more likely to develop HF compared to women without diabetes [20].

DC is a disease which does not have a consistent definition despite being known since 1972 when Rubler et al. first described it. In DC, we can observe cardiac dysfunction in the absence of hypertension, coronary artery disease and valvular disease. Moreover, in the pathogenesis of DC, there is a huge role of multiple metabolic disturbances, such as: glucose toxicity and lipotoxicity, microcirculatory disorder, vascular endothelial dysfunction and capillary failure [20].

### 2.1. Epidemiology 

HF is the most common cardiovascular complication in patients with DM. First indications of cardiomyopathy can be seen in pre-DM patients with metabolic syndrome. Even a 1% rise in baseline level of HbA1c results in a 15% increase in risk of HF development both in patients with and without DM. The risk is higher in T1DM at 30% and significantly lower in T2DM, where it stands at 8%. On the other hand, the reduction in HbA1c levels can lower the risk of HF development. Patients with idiopathic cardiomyopathy often have underlying DM. The duration of DM has an influence on the prevalence of left ventricle (LV) dysfunction. Due to more advanced echocardiographic techniques, in recent studies on DC, there is more highlight on diastolic dysfunctions in comparison to more outdated ones describing mostly contractile dysfunctions [9,21,22,23].

### 2.2. Pathophysiology 

**Figure 1:**
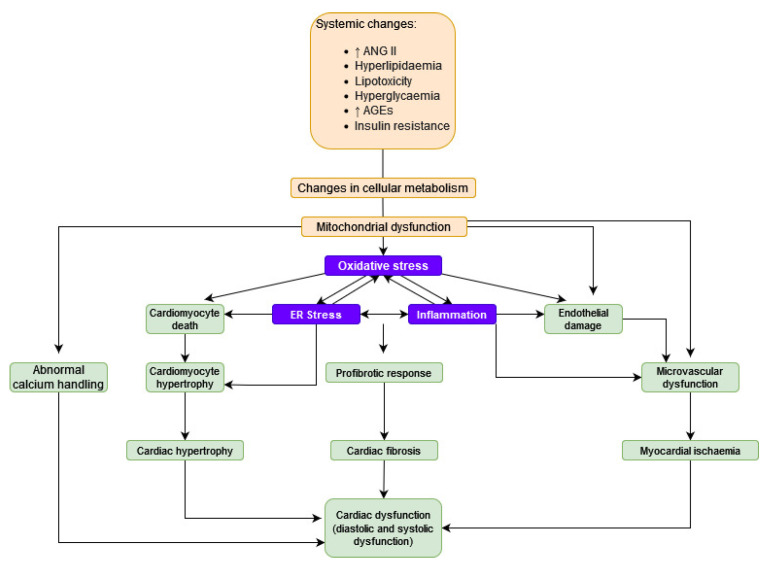
The pathophysiology of DC.

Pathogenesis of cardiomyopathy associated with diabetes is multifactorial. Among them, the most important ones include: lipotoxicity, oxidative stress, mitochondrial dysfunction, inflammation, abnormal myocardial calcium handling and autonomic dysregulation in the heart. Molecular abnormalities induced by DM differ from those resulting from hypertension or ischemia [20,21,23,24]. The pathophysiology of DC is presented in Figure 1. 

#### 2.2.1. Lipotoxicity

In DC, there is a dysfunction in cardiac metabolism due to lipotoxicity, which is depicted in Figure 1. Increased levels of free fatty acids (FFA) in the blood result in the accumulation of FFA in cardiac cells. In the myocardium, FFA beta-oxidation is used to produce energy, yet it is a less effective mechanism than using carbohydrates as an energy substrate. As a result, it decreases the efficiency of the cardiac tissue.

Excessive FFA are turned into:

- Diacylglycerol, which leads to exacerbation of insulin resistance and oxidative stress by activation of protein kinase C (PKC);
- Ceramide, which leads to oxidative stress and the dysfunction of mitochondria.

Furthermore, peroxisome proliferator-activated receptor α (PPAR α) is activated by FFA, which results in increased uptake via CD36 [9,20,21,23].

#### 2.2.2. Oxidative Stress

Oxidative stress (OS) is a well-established cause for the development and progression of DC and its complications, as it is one of the major factors responsible for causing cardiac fibrosis and hypertrophy. Excessive OS is promoted by hyperglycaemia and occurs in DM through several mechanisms, such as:

- Abnormal regulation of the electron transport chain in mitochondria;
- Increased nicotinamide adenine dinucleotide phosphate (NADPH) oxidase activity;
- Influence of advanced glycation end products (AGEs)

Reactive oxygen species (ROS) are able to mutilate proteins or phospholipids by direct oxidation, or secondarily by oxidising lipids (reactive lipid peroxides) or by creating ROS from nitric oxide (NO) [20,24]. Advanced glycation end products (AGEs) are also linked with oxidative stress. Cardiotoxic effects of AGEs do not result from their direct action, but rather depend on an increase in ROS production. Due to AGEs influence, nicotinamide adenine dinucleotide phosphate (NADPH) oxidase activity is enhanced, which leads to increases in oxidative stress and inflammation. They also cause cross-linking of collagen molecules to each other. Thus, AGEs lead to contractile dysfunction by loss of collagen elasticity. In high glucose levels, Nf-κB mediated inflammatory signalling is activated, while Nrf2- and Sirt1-mediated antioxidant signalling is inhibited. As a result of those changes, the production of ROS and inflammatory factors increases. The aforementioned factors accelerate pathological changes in cardiac tissue, such as remodelling and fibrosis, and therefore lead to cardiac dysfunction [9,20,21].

#### 2.2.3. Abnormal Calcium Handling

Disturbances in cellular energetic metabolism also affect proper functioning of intra- and extracellular Ca^2+^ pumps, which result in increased Ca^2+^ level inside the cardiomyocytes and disrupt calcium handling and signalling. The causes of impaired calcium homeostasis may be the damage to the ryanodine receptors (RyR), which are responsible for releasing calcium from the sarcoplasmic reticulum (SR), caused by oxidative stress. Impaired insulin signalling also disrupts functioning of insulin-stimulated coronary endothelial nitric oxide synthase (eNOS) activity, which results in decreased nitric oxide (NO) production and therefore increases cardiomyocyte intracellular calcium sensitization and reduces sarcoplasmic Ca^2+^ uptake. Calcium is an essential mediator of cardiac contractile and diastolic functions as well as its electrical stability; thus, impaired calcium homeostasis promotes arrhythmias and mechanical dysfunctions, especially diastolic ones characteristic for DC [8,9,20,21].

### 2.3. Clinical Picture 

The onset of DC is insidious, as it may not produce symptoms for a long time, despite the progression of myocardial damage. First, echocardiographic abnormality detected in the early phases is left ventricular diastolic dysfunction with thickening of the left ventricle wall, which may develop into HFpEF. As the disease progresses, left ventricular systolic dysfunction may deteriorate, often resulting in heart failure with reduced ejection fraction (HfrEF) [20,21,25]. Progression of DCM can be divided into three stages: early, advanced, and late, which is presented in Table 1 [20,25].

### 2.4. Prognosis 

Unfortunately, the diagnosis of DC is usually set when the disease is symptomatic and the left ventricle ejection fraction (LVEF) is already lowered. Diabetic patients who develop HF are treated according to the same treatment regimen as non-diabetic ones. However, T2DM and HF as comorbidities are associated with 33% greater hospitalisation rate and faster progression of the disease. At this moment, there is no accurate screening test available and no clear diagnostic criteria for DC, yet there is a great need for further research [21,25].

### 2.5. Therapeutical Grip Points

Prevention and treatment of DC aim to control the components of the underlying disorder relevant to the pathogenesis of the disease, such as insulin resistance, impaired lipid levels, excessive inflammation and oxidative stress [23]. Classic hypoglycaemic drugs such as metformin are used in treatment, but a very promising therapeutic option is the new generation of drugs, above all SGLT-2i, due to their cardioprotective properties [20,23].

## 3. SGLT-2is as a Drug Class

SGLT-2is are on the cutting edge of medical discoveries in the 21st century, as they have completely revolutionised the pharmacotherapeutic regimen not only for diabetes, but also for heart failure and chronic kidney disease. Although these specific molecules were first introduced in 2005 in a study on dapagliflozin conducted by Zhang et al., a natural, non-selective SGLT inhibitor called phlorizin had been studied for over 200 years, yet only recently have scientists discovered its mechanism of action [26,27]. Florisin is a natural flavonoid isolated from the bark of apple trees, and as early as in the 19th century was it observed to cause glucosuria and lower blood glucose levels [28,29]. Florisin use has been limited due to poor intestinal absorption and numerous side effects, yet it was the lead compound in understanding the physiology of SGLT and designing research on modern drugs targeting SGLT2 receptors [30,31]. This research has culminated in FDA approval of canagliflozin (marketed as Invokana^®^) in March 2013, followed by the approval of dapagliflozin (marketed as Forxiga^®^) in January 2014 and empagliflozin (marketed as Jardiance^®^) in August 2014. Initially, these drugs were registered as antidiabetic agents, but the pleiotropic effects of flozins were promptly observed and became the field of extensive studies such as EMPA-REG OUTCOME, CANVAS Program, SCORED and DECLARE-TIMI. These studies have established the beneficial effects of SGLT-2is not only on glycaemic control but also on the cardiovascular system and patient quality of life [32]. Currently, SGLT-2is are the only drugs with proven efficacy in the management of HF in every range of ejection fraction according to the EMPEROR-REDUCED, EMPEROR-PRESERVED, DAPA-HF, DELIVER studies, and they have been incorporated in recent HF treatment algorithms [33,34,35,36]. Apart from HF management, in August 2021, dapagliflozin was approved in the European Union for chronic kidney disease [CKD] treatment regardless of diabetic status owing to the results of the phase III DAPA-CKD trial [37,38]. However, numerous studies such as EMPA-KIDNEY and EMPULSE are being conducted to investigate the pleiotropic properties of flozins; therefore, the potential of this drug class has not yet been exploited [39].

There are two types of SGLT cotransporters: SGLT-1 present in the kidney, heart and intestine, where it is majorly expressed, participating in the glucose and galactose absorption in the gastrointestinal tract, and SGLT-2 specific to the kidney [27]. SGLT-2 is expressed in the renal proximal tubules and is responsible for glucose reabsorption from the glomerular filtrate, and then, reabsorbed glucose is transported by GLUT2 and GLUT1 receptors back to the bloodstream [27,40]. Physiologically, the kidneys filter approximately 180 L of plasma every day, containing about 5·5 mmol/L of glucose, compatible with 180 g of glucose filtered, almost all of which is reabsorbed [40]. In diabetic patients, there is an overexpression of the SGLT-2 protein, which leads to increased glucose renal reabsorption, thus contributing to hyperglycaemia [41]. Flozins through inhibiting SGLT-2 provide lower glucose reabsorption in the kidneys and therefore lower glycaemia, which is an efficient blood glucose control mechanism totally independent from the insulin hormone [41]. Such a mechanism of action makes these drugs useful in both monotherapy and in association with other antidiabetic drugs and suitable to be implemented in different evolutionary stages of the disease [41]. Another beneficial feature of this medication group is their safe profile of use and an oral administration with an accessible dosage regimen. The most common side effects are genitourinary infections, yet in most cases, they are easily preventable through proper hygiene and patient education [37,42]. However, there are also rare, but serious side effects which also may occur such as euglycaemic diabetic ketoacidosis (DKA) [37].

**Figure 2:**
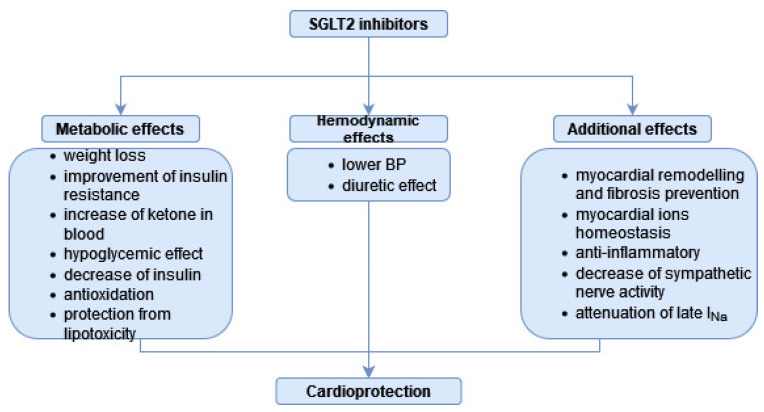
Mechanism of cardioprotection displayed by SGLT-2is.

As the effects of SGLT-2is extend widely beyond glycaemic control, they provide a more holistic treatment of both diabetic and non-diabetic patients. SGLT-2is have shown significant effects in reducing CV mortality, atherosclerotic CV disease complications and in minimising hospitalizations in chronic HF patients as well as significantly improving their quality of life [43,44,45,46,47]. The cardioprotective mechanism of SGLT2-is is depicted in Figure 2. Flozins owe their cardioprotective properties to their effects on haemodynamic parameters such as preload by reducing plasma volume through increased diuresis and decreasing plasma sodium content, and on afterload by lowering arterial pressure and reducing the degree of arterial stiffness [32,48,49,50]. In addition, flozins have demonstrated a beneficial effect in inhibiting myocardial remodelling and fibrosis and in improving contractile function [32,51]. These properties are most likely attributed to the effect of SGLT-2is on Na^+^/H^+^ exchanger 1 (NHE1) in cardiomyocytes, influencing homeostasis of intracellular sodium and calcium ion levels, which ensures adequate contractile function, resistance to oxidative stress and reduction of arrhythmias [51,52,53]. Despite the absence of SGLT-2 receptors in the heart, this mechanism of action has been demonstrated for empagliflozin, dapagliflozin and canagliflozin; therefore, it has been identified as a class effect [32]. SGLT-2 inhibitor drugs also have an impact on CV risk factors by affecting weight loss and lipidogram and by lowering serum uric acid levels [50,54]. Several studies reported lowering of total cholesterol [TC] and triglycerides [TGs], and increases in both high-density lipoproteins [HDLs] and low-density lipoproteins [LDLs] levels during SGLT-2 treatment [55,56]. Despite causing an increase in LDL-levels, SGLT-2is maintain their cardiovascular and renoprotective effect owing to the increase in large, floating LDL particles with lower atherogenic potential than small, dense, highly inflammatory ones [57,58]. Renoprotective properties of SGLT2is have been demonstrated through reducing albuminuria and slowing down the progression of chronic kidney disease. The mechanism of these effects is multifactorial, including: reduced arterial and intraglomerular pressure, diminished renal hyperfiltration, increased natriuresis, reduction in uric acid levels and anti-inflammatory effects [32,42]. Characteristic for this drug class is a bi-phasic effect on eGFR with a temporary decrease after introducing flozins into treatment followed by long-term eGFR stabilisation [37,42]. There has been growing evidence that suggests SGLT2is to have a neuroprotective potential. In a study conducted by Hierro-Bujalance et al. on a mouse mixed model of diabetes mellitus and Alzheimer’s disease, empagliflozin improved both cerebral microvascular functions and cognitive impairment [59]. Flozins may bring neuroprotective effects through several mechanisms, including their anti-inflammatory and anti-atherosclerotic properties, reduction in oxidative stress, amelioration cerebrovascular remodelling and restoring a balance between catabolism and anabolism [59,60].

Owing to this their pleiotropic effects, SGLT-2is have also been studied in patients with type 1 diabetes mellitus (T1DM) in phase 3 clinical trials such as the inTandem with sotagliflozin, DEPICT using dapagliflozin, and EASE investigating empagliflozin. Primary results of these trials demonstrated consistent reductions in insulin doses and HbA1c levels in the SGLT-2 group, yet with higher risk of diabetic ketoacidosis (DKA) compared to placebo [61,62,63]. Secondary analyses of mentioned trials have also revealed potential nephroprotective effects of flozins that are independent of improved glycaemic control [64]. However, the study conducted by Komoshima et al. revealed that adding SGLT2is to intensive insulin treatment in patients with T1DM improves glycaemic control and body mass without increasing the incidence of DKA and hypoglycaemic events [65]. Additional research is required to determine nephro- and cardioprotective effects of SGLT-2is added to insulin treatment and whether these beneficial effects exceed the risk of DKA. In addition, there is a necessity to assess the efficiency of DKA preventative measures such as patient education and ketone monitoring in reducing the risk of such a complication during flozin treatment in T1DM [64].

## 4. SGLT-2is in DC—The Overview of Research Results

SGLT-2is, despite their relatively short presence in the pharmaceutical market, have quickly become highly desirable players in the therapeutic area. Besides the SGLT-2is direct hypoglycaemic effects awaited in DM therapy, they have been reported to present remarkable cardio- and renoprotective properties [66]. Considering the frequent co-occurrence of T2DM and cardiovascular diseases, flosins are of great interest due to their broader spectrum of indications for prospective use. In CV, the benefits of flozins are derived not only from their primary anti-diabetic mechanism of action, but from an indirect activity as well. Many clinical and experimental studies demonstrate the efficacy of SGLT2i in preventing cardiovascular complications. For instance, the 5-year cumulative incidence risk among the 2849 patients who had met the criteria for both EMPA-REG-like and DECLARE-like was reduced from 97.1 to 75.6 cardiovascular incidents through SGLT2is [67]. Based on the SAVOR-TIMI 53 and DECLARE-TIMI 58 trial results, SGLT2is were assessed to reduce HF risk to a greater degree in patients at high and very high risk (respectively, 1.5% and 2.7% absolute reductions of hospitalization for heart failure risk at 4 years) [68]. Following CVD-REAL study analysis, introduction of SGLT-2is in comparison to other glucose-lowering agent treatments was estimated with a moderate reduction of risk of myocardial infarction (MI) and stroke (MI: HR, 0.85; 95%CI, 0.72–1.00; *p* = 0.05; Stroke: HR, 0.83; 95% CI, 0.71–0.97; *p* = 0.02) [69]. Furthermore, in CVD-REAL Nordic, dapagliflozin lessened the risk of major adverse cardiovascular events by 0.79 (95% CI, 0.67–0.94), HF 0.62 (95% CI 0.50–0.77) and all-cause mortality 0.59 (95% CI 0.49–0.72) as compared to dipeptidyl peptidase inhibitors (DPP-4i) [70]. Dapagliflozin was also clinically assessed to reduce HbA1c, weight and SBP in patients with T2DM and HF [71]. It was proven that HbA1c can be significantly reduced with canagliflozin [72] or tofogliflozin [73] as well. Following in vivo studies in mice, dapagliflozin showed a hypoglycaemic effect after 8 or 12 weeks of treatment and reduced insulin, triglyceride and cardiac BNP levels [74,75,76]. As for the results of the EMPA-HEART CardioLink-6 trial (Effects of Empagliflozin on Cardiac Structure in Patients with Type 2 Diabetes) in patients with T2DM and coronary artery disease, 6-month empagliflozin (10 mg/day) treatment revealed potential drug-induced regression of left ventricle hypertrophy. Altogether, reduced systolic and diastolic blood pressure, an increase in hematocrit, although no change in NT-proBNP levels nor LV volume, were observed. Improvement in the LV mass index was statistically assumed to be independent of antihypertensive effect despite the correlation [77]. Carbone et al. hypothesised a synergistic effect of cotreatment with empagliflozin and loop diuretics that could relieve cardiac preload [78]. Not surprisingly, the effect of empagliflozin treatment expressed in ventricular functioning improvement is preferred in patients without developed HF in the early diabetic cardiomyopathy stages [79].

Multiple studies on SGLT2i were conducted in mice models of DC. In diabetic genetically mutated mice in which leptin receptors do not function properly (db/db mice), reduced mortality and improved LV diastolic function were observed after 4.5-week empagliflozin treatment [80]. Enhanced ventricular functions were associated with increased AKT-phosphorylation, which may be responsible for better insulin sensitivity [80]. Meanwhile, in homozygotus, genetically obese, leptin-deficient (ob/ob^−/−^) mice, improvement of coronary microvascular function and contractility as well as better lipid profile were reported after 10-week-long empagliflozin treatment [81]. Similarly, in Wistar rats, 8-week-long empagliflozin treatment showed hypoglycaemic activity and improved cardiac functions with reversed histopathological changes of myocardium [82]. In a study conducted by Li et al., empagliflozin was proven to decrease transforming growth factor alpha 1 (TGFβ1), which promotes heart remodelling and cardiomyofibrosis, by 73.2% as compared to the control group [83]. Cardiac electromechanical effects of empagliflozin treatment showed reversed diabetes-associated cardiac hypertrophy as well as electrophysiological changes in rodent heart such as prolongation of QT and ventricular action potential [84]. In addition, ertugliflozin and canagliflozin efficiently improved left ventricular function and reduced myocardial fibrosis [72,85].

More molecular studies confirmed that empagliflozin ameliorates the expression of cardiac hypertrophy markers such as actin alpha (ACTA1), four-and-a-half LIM domains 1 (FHL1), myosin light chain 2a (MLC2A) in high-glucose-exposed human-induced pluripotent stem cells-derived cardiomyocytes [66]. In addition, SGLT2is cardioprotective effects have been linked to activation of the Janus-activated kinase-signal transducer and activator of transcription (JAK2/STAT5), phosphatidylinositol-3-kinase-Akt (PI3K/Akt), and extracellular signal-related kinase (ERK/MAPK) signalling pathways, as they are associated with proliferation and differentiation of cardiomyocytes. El-Sayed et al. suggested that mentioned signalling pathways interact with erythropoietin activity, which could modulate cardioprotective effects of dapagliflozin [86]. Furthermore, in silico analyses predicted the interference of dapagliflozin with AGE-RAGE, TNF and MAPK signalling pathways associated with the development of DC [87]. Empagliflozin and canagliflozin target MAPK signalling as well [87]. An elevated ratio of phosphorylated signal transducer and activator of transcription 3/signal transducer and activator of transcription 3 (pSTAT3/STAT3) protein expression was consecutively presumed to be antifibrotic [88]. Moreover, Xue et al. pointed out the importance of the guanylate cyclase-cyclic guanosine monophosphate-protein kinase G (GC-cGMP-PKG) pathway, which mediated the potency of empagliflozin to alleviate diabetes-induced oxidative injury, apoptosis and pyroptosis [89]. This pathway is repressed in diabetes, and SGLT2is restore its activity [89]. Summarising all of the above, SGLT2is provide a great benefit in DC by preventing or even reversing diabetes-induced cardiac remodelling.

The effect of SGLT2is on metabolism also has significance in DC. An altered mitochondrial metabolism in T2DM may result in cardiac lipotoxicity, insufficient energetic supplies and further progression to heart failure. Marfella et al. conducted a study on heart transplants. Curiously, in diabetic recipients, there was observed lipotoxic cardiomyocyte dysfunction associated with peroxisome proliferator-activated receptors-g (PPAR-g) overexpression, which could be reduced with the SGLT2is [90]. In a recent study conducted by Zhang et al., empagliflozin targeted the mitochondrial fatty acid oxidation pathway, which is altered in db/db mice hearts [91]. SGLT2i has been reported to induce phospho-AMP-activated protein kinase alpha 2 (pAMPKalpha2), which is responsible for metabolic switching from fatty acids and maintenance of energy balance [88]. Similarly, in insulin-resistant mice, 4-week empagliflozin treatment was proven to be bioenergetically beneficial, as it restored cardiac metabolic flexibility regarding metabolic substrate selection and promoted glucose utilisation. Thus, it counteracted ventricular hypertrophy and ischemia-related stress [92]. Moreover, the treatment improved overall cardiomyocytes ATP production when compared to diabetic mice, which was provided via an increase in the rate of glucose to fatty acids oxidation, yet there was no significant change in the rate of ketone oxidation [92,93]. Energetic benefits were also detected in the 18-patient cohort after 12 weeks of empagliflozin treatment, as there were significant improvements in the phosphocreatine-to-ATP ratio and a concomitant 7% increase in the mean LVEF [94].

SGLT2is can also influence the function of enzymes that are involved in the metabolism of toxic metabolites. In DC, a lower activity of aldehyde dehydrogenase 2 has been detected, which, as a consequence, increases lipid peroxidation, resulting in elevated levels of reactive aldehyde 4-hydroxy-2-nonenal (4HNE). A study on aldehyde dehydrogenase 2 (ALDH2) mutant diabetic mice subjected to 2-month 3 mg/kg/d empagliflozin treatment resulted in an improvement in exercise performance and cardiac function with reduced cardiac mitochondrial 4HNE; therefore, empagliflozin implementation reversed the effects of ALDH2-impaired activity [95,96].

Another important issue considering DC is the positive influence of flozins on the endothelium [97]. Ipragliflozin was capable of attenuating endothelial dysfunction in streptozotocin-induced C57BL/6 mice considering its anti-inflammatory and antioxidative properties [98]. In Zucker diabetic fatty-Leprfa/fa rats, empagliflozin treatment as long as 6 weeks was proven to protect the endothelium, likely owing to the spectrum of hypoglycaemic effects including inhibition of AGE/RAGE axis, preventing oxidative stress and inflammation [96]. Its vasoprotective effect, including vasorelaxation, was assessed to be mediated by NO [81,96]. Analogous conclusions considering empagliflozin and its protective effects on the endothelium were drawn for human vascular endothelial cells investigated by Mone et al. [99].

SGLT2is have the ability to regulate ion homeostasis, especially myocardial Na^+^ and Ca^2+^ concentration. Flozins attenuate increased cardiac late Na current (INa-late) and NHE currents, prevent decreased Ca^2+^ transients and Ca^2+^ stores as well as restore decreased NCX (Na^+^/Ca^2+^ exchange) current. These properties have brought, to a further conclusion, possible antiarrhythmic properties of SGLT2is [84]. Moreover, SGLT2is decrease calmodulin-dependent protein kinase II (CaMKII) activation while inhibiting RyR(Ser2808)-phosphorylation [80]. CaMKII expression is promoted by ROS, and its chronic activation is involved in OS-mediated heart dysfunctions [100]. They also contribute to the attenuation of the voltage-dependent L-type calcium channel (CACNA1C) and NCX and NHE membrane transporters expression, which prevents intracellular Na^+^ and Ca^2+^ loading [101]. Furthermore, SGLT2is alleviate vascular permeability and mitochondrial Ca^2+^ overload [99]. According to the study of Osaka et al., hyperglycaemic-stimulated NHE-1 activity was reversed via luseogliflozin in mice cardiomyocytes, which resulted in the attenuation of TGF-β2 expression and mitigation of cardiomyocyte hypertrophy and fibrosis [102].

Excessive inflammation and OS are key factors in the pathomechanism of DC. SGLT2is demonstrate anti-inflammatory activity by suppressing NOD-like receptor 3 (NLRP3) activation and downregulating TNFα, IL-1β, IL-6, FGF21 and caspase-1. This mechanism also involves the activation of myocardial AMPK and mTOR complex 2 (mTORC2) [74,75,85,88,103]. A study by Hussein et al. revealed the reduction in levels of inflammatory TNF-α, TGF-β cytokines and cardiac caspase-3 after 4 weeks of dapagliflozin exposure, alongside the attenuation of sympathetic activity and downregulation of myocardial tyrosine hydroxylase activity [104]. In human cardiomyocytes cultures, empagliflozin significantly reduced the release of the interferon (IFN)γ-induced 10 kDa protein (IP-10/CXCL10), a chemoattractant associated with inflammation and development of cardiomyopathy. Additionally, empagliflozin downregulated the activation of Stat-1, a signal transducer, which is also involved in cardiac remodelling [105]. Another proinflammatory factor relevant to DC pathogenesis is tumour necrosis factor receptor-associated factor 3 interacting protein 2 (TRAF3IP2), a proinflammatory protein, which was found to be elevated in H9C2 cardiomyocytes under chronic hyperglycaemia. Furthermore, TRAF3IP2 overexpression is linked to oxidative stress, chronic inflammation and fibrotic proliferation in the heart. Hypoglycaemic agents, including empagliflozin, exhibited the ability to downregulate the expression of TRAF3IP2 in a dose-dependent manner, presenting at the same time cardioprotective properties [106]. In diabetic rats and high glucose-stimulated rat embryonic cardiac myoblast H9C2 cells, both undergoing dapagliflozin treatment, the favourable mechanism involved a decrease in copper zinc superoxide dismutase (Cu/Zn-SOD) expression, which resulted in attenuating myocardial fibrosis and higher cell viability [107]. Furthermore, Wistar rats with metabolic syndrome treated with dapagliflozin for 2 weeks showed an improvement in cardiomyocyte function through affecting Zn^+2^-transporters ZIP7, ZIP14, ZIP8 and ZnT7, thus benefiting total oxidative status [108]. Joubert et al. investigated the influence of dapagliflozin on diabetic lipodystrophic Bscl22/2 SKO mice and observed a significant decrease in glucose overload-triggered levels of O-GlcNAcylated proteins, in particular forkhead box protein O1 (FOXO1) [109]. Another antioxidative property presented by empagliflozin involves suppressing NOX4 and attenuating oxidation products. The study by Wang et al. showed an intensified expression of Nrf2 and HO-1 protein, which was impaired in non-treated diabetic groups, and the promotion of Nrf2 nuclear translocation in the empagliflozin group [83,110]. Wang et al. also described cardio-protective effects of empagliflozin treatment studied in both db/db mice and palmitate-exposed H9C2 cardiomyocytes, which include attenuating of cardiac remodelling, increasing sensitivity to insulin and supporting mitochondrial activity. The ability to promote Nrf2 is one of the factors that determines antioxidative properties of empagliflozin and, overall, stands for a compound process of preventing diabetic cardiomyopathy [110].

Of major importance to diabetic patients is the effect of SGLT-2 inhibitors on the course of cardiovascular complications to which they are particularly predisposed, such as acute coronary syndromes, especially acute myocardial infarction (AMI). According to data from an ongoing observational registry: SGLT2-I AMI PROTECT patients that had been administered SGLT-2is prior to percutaneous coronary intervention (PCI) exhibited significantly reduced inflammatory response and smaller infarct size compared to those receiving other oral hypoglycaemic agents [111]. In the retrospective analysis conducted by Zhu et al., patients treated with dapagliflozin after AMI had a significantly lower rate of major adverse cardiovascular events, including overall deaths, HF, MI, stroke and unplanned repeat revascularization compared to the DAPA-free group [112]. According to the EMMY trial results introducing 10 mg empagliflozin once daily for patients that underwent recent AMI with PCI, it is associated with a significantly greater NT-proBNP reduction over 26 weeks, accompanied by a significant improvement in echocardiographic parameters [113]. Although the long-term benefits of SGLT-2is are particularly compelling, the Baker et al. study [114] demonstrated that their short-term effects may also determine favourable treatment outcomes. In this study, a series of haemodynamic measurements were performed on swine models administering a 15–30 min infusion of either canagliflozin or the NHE-1 inhibitor cariporide immediately before the occlusion of the left circumflex artery. Pre-ischemic treatment with canagliflozin resulted in a significant increase in both diastolic filling and stroke work and improved cardiac work efficiency in comparison to untreated control hearts during ischemia. In swine models treated with cariporide, there were no such favourable effects, which suggest that SGLT-2is has beneficial effects that are not associated with NHE-1 inhibition in this particular situation. Therefore, this short-term effect may provide protection against ischemia in the context of chronic treatment.

## 5. Conclusions

SGLT-2is represent a breakthrough in the treatment of not only T2DM as initially intended, but also other conditions such as HF and CKD regardless of codominant T2DM. Designing the treatment for a patient with T2DM, SGLT-2is provide the chance to manage this disorder holistically by targeting not only glycaemic control, but also by acting in a cardio- and renoprotective manner, therefore preventing T2DM complications. In view of the reported neuroprotective effect of SGLT-2 inhibitors in patients with Alzheimer’s disease, it is strongly believed that the considerable potential of this group of drugs still remains undiscovered, and it will remain a hot scientific topic for a long time to come. In our article, we focused on the T2DM complication DC and aimed to establish the position of flosins in managing this disorder. SGLT-2is act in a multifaceted way to modulate many pathophysiological aspects of DC. Representatives of SGLT-2is have been proven to attenuate inflammation and OS, and positively impact myocardial ion homeostasis and energetic balance, thereby preventing cardiac remodelling, fibrosis and hypertrophy and thus preventing left ventricular systolic and diastolic function, which are primary DC components. Studies on mice models have shown that SGLT-2is, especially empagliflozin, potentially prevent and even reverse diabetes-induced histopathological changes of the myocardium. These referred properties are particularly beneficial in the early stages of the disease and therefore suggest the relevance of implementing SGLT-2is as early as the diagnosis of diabetes is set in order to maximise the cardioprotective effects of this drug class and minimise the risk of developing complications. As for complications, SGLT-2 therapy not only reduces the incidence of acute cardiovascular incidents, but also helps to mitigate their dramatic course by decreasing the size of the infarction. However, the beneficial effects of flozins extend to more advanced cases of DC with present LV dysfunction as well as symptomatic HF, as SLGLT-2is are the only drugs with proven benefit in heart failure in patients of any LVEF spectrum. Therefore, implementing SGLT-2is into treatment at any stage of DC seems particularly beneficial, not to mention the safe profile of flozins and their rare and preventable side effects. The favourable profile of these drugs, confirmed by numerous scientific reports, should persuade a change in treatment algorithms for carbohydrate metabolism disorders towards flosins being listed as a first-line drug, not only in the case of comorbid cardiovascular or renal disease, but in each patient with a novel diagnosis. However, the price and reimbursement regulations also remain an issue in many countries, as the attempt to introduce these drugs into treatment may often be met with patient refusal due to economic reasons. Consequently, there is a growing need for further, specific research on SGLT-2is benefits in DC as well as on their use in DMT1, during the peri-operative period and the safety of flozins use in patients prone to some of their adverse effects.

## Figures and Tables

**Table 1:** Progression of DC.

Stage	Pathophysiological Events	Changes in Structure and Morphology	Functional Impairment
Early	Hyperglycaemia, downregulation of GLUT4, insulin resistance, increase in free fatty acids, impairment of Ca^2+^ homeostasis, sympathetic nervous system activation	Small pathophysiological changes in cardiomyocytes, normal left ventricular mass and LV wall thickness	Little or no left ventricular diastolic dysfunction (LVDD)
Advanced	Cardiomyocyte injury and death, fibrosis, activation of RAAs, increased inflammation	Increased left ventricular mass, wall thickness and size	Impairment of left ventricular diastolic function and slightly decreased ejection fraction
Late	Myocardial fibrosis, impaired microvascular coronary circulation, severe neurohormonal activation, inflammation	Substantial increase in left ventricular mass, wall thickness and size	Impairment of both diastolic and systolic functions
